# Quantifying the Relative Contributions of Divisive and Subtractive Feedback to Rhythm Generation

**DOI:** 10.1371/journal.pcbi.1001124

**Published:** 2011-04-21

**Authors:** Joël Tabak, John Rinzel, Richard Bertram

**Affiliations:** 1 Department of Biological Sciences, Florida State University, Tallahassee, Florida, United States of America; 2 Courant Institute of Mathematical Science and Center for Neural Science, New York University, New York, New York, United States of America; 3 Department of Mathematics and Programs in Neuroscience and Molecular Biophysics, Florida State University, Tallahassee, Florida, United States of America; École Normale Supérieure, College de France, CNRS, France

## Abstract

Biological systems are characterized by a high number of interacting components. Determining the role of each component is difficult, addressed here in the context of biological oscillations. Rhythmic behavior can result from the interplay of positive feedback that promotes bistability between high and low activity, and slow negative feedback that switches the system between the high and low activity states. Many biological oscillators include two types of negative feedback processes: divisive (decreases the gain of the positive feedback loop) and subtractive (increases the input threshold) that both contribute to slowly move the system between the high- and low-activity states. Can we determine the relative contribution of each type of negative feedback process to the rhythmic activity? Does one dominate? Do they control the active and silent phase equally? To answer these questions we use a neural network model with excitatory coupling, regulated by synaptic depression (divisive) and cellular adaptation (subtractive feedback). We first attempt to apply standard experimental methodologies: either passive observation to correlate the variations of a variable of interest to system behavior, or deletion of a component to establish whether a component is critical for the system. We find that these two strategies can lead to contradictory conclusions, and at best their interpretive power is limited. We instead develop a computational measure of the contribution of a process, by evaluating the sensitivity of the active (high activity) and silent (low activity) phase durations to the time constant of the process. The measure shows that both processes control the active phase, in proportion to their speed and relative weight. However, only the subtractive process plays a major role in setting the duration of the silent phase. This computational method can be used to analyze the role of negative feedback processes in a wide range of biological rhythms.

## Author Summary

As modern experimental techniques uncover new components in biological systems and describe their mutual interactions, the problem of determining the contribution of each component becomes critical. The many feedback loops created by these interactions can lead to oscillatory behavior. Examples of oscillations in biology include the cell cycle, circadian rhythms, the electrical activity of excitable cells, and predator-prey systems. While we understand how negative feedback loops can cause oscillations, when multiple feedback loops are present it becomes difficult to identify the dominant mechanism(s), if any. We address the problem of establishing the relative contribution of a feedback process using a biological oscillator model for which oscillations are controlled by two types of slow negative feedback. To determine which is the dominant process, we first use standard experimental methodologies: either passive observation to correlate a variable's behavior to system activity, or deletion of a component to establish whether that component is critical for the system. We find that these methods have limited applicability to the determination of the dominant process. We then develop a new quantitative measure of the contribution of each process to the oscillations. This computational method can be extended to a wide variety of oscillatory systems.

## Introduction

Biological systems involve a large number of components that interact nonlinearly to produce complex behaviors. How can we determine the role that a component plays in producing a given behavior of the system? We approach this question in the relatively simple context of relaxation oscillations, since relaxation oscillator models and their extensions are used to describe a wide variety of biological behaviors [1], such as the cell cycle [2], electrical activity of cardiac and neural cells [3], [4], circadian patterns of protein synthesis [5], metabolic oscillations [6] and episodic activity in neuronal networks [7]. Specifically, we use a model developed to describe the rhythmic activity of developing neural networks and whose formalism also applies to cellular pacemakers [8]. The activity of the system can be either high or low, and slow negative feedback processes switch the system back and forth between the active and silent states. Hence the rhythm consists of episodes of high activity separated by silent phases, repeated periodically. While relaxation oscillator models usually contain one negative feedback process to regulate the rhythmic activity, in biological systems two or more feedback processes are often present. Thus, we consider a model with two different types of negative feedback: divisive and subtractive. In the context of an excitatory network, synaptic depression (weakening of synaptic connections between neurons) is a divisive feedback (decreasing the slope of the network input/output function) while activation of a cellular adaptation process (decreasing the neurons' excitability) can be a subtractive feedback (shifting the network input/output function) [8], [9]. With both types of negative feedback in the model, we seek to determine the contribution that each makes to episode initiation and termination.

We begin by using two strategies based on the two broad types of experimental protocols. The correlative strategy seeks to detect associations between the time course of a variable and the system's behavior. To use the example of episodic activity generated by an excitatory neural network, we compare the variation of the fraction of undepressed synapses (weighted by the synaptic conductance) to the activation of the cellular adaptation current (scaled by its conductance). Intuitively, the process that shows the greatest changes would be considered to affect activity the most, and thus contribute more to episode initiation/termination. The faster process covers a wider range during the active and silent phases [8]. This predicts that the faster a process and the larger its associated weight, the more it contributes to episode initiation and termination. The second strategy is to block one process, with the expectation that this will result in changes in activity that are directly related to the contribution of that process. Perhaps contrary to intuition, we find that blocking a slow process may provide little information on the role of that process in the rhythm generation, and that the correlative and blocking strategies may even lead to contradictory conclusions.

We then develop a new strategy based on the idea that if a negative feedback process contributes significantly to episode termination, then increasing its time constant should significantly increase episode duration. Similarly, if recovery of such a process contributes to episode initiation, then increasing its time constant should significantly delay episode initiation. We develop a measure of the respective contribution of each process based on these ideas. This measure reveals that if the divisive and subtractive feedback processes have similar time scales and similar weight they contribute similarly to episode termination. In contrast, the subtractive process controls episode initiation, even if it is slower or has less weight. This also means that the divisive process only plays a minor role in episode initiation. This unexpected result was not revealed using the standard approaches, and demonstrates the utility of the new measure in pulling out the key dynamics involved in rhythm generation. These results demonstrate that the characteristics of the correlative and blocking methods limit their usefulness in the determination of which feedback process controls rhythmic activity. Instead, this question requires computational tools such as the ones developed here. Finally, we point out in Discussion that hybrid systems such as the dynamic clamp may allow experimental use of our method.

## Model

We consider a mean field type model describing the activity of an excitatory neural network subject to both synaptic depression and cellular adaptation as described previously (Tabak et al., 2006). The variables of the model are *a*, the network activity (firing rate averaged across population and time; *a* = 0 corresponds to all cells silent, *a* = 1 means all cells fire at their maximal frequency); *s*, the fraction of undepressed synapses (*s* = 0 means all synapses are depressed, *s* = 1 means all synapses are operational); and *θ*, a cellular adaptation process that raises the neuronal firing threshold (*θ* = 0 means no adaptation so the cellular threshold is at its baseline level θ_0_, *θ* = 1 is the maximal adaptation). The model equations are:
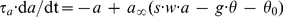
(1)

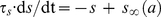
(2)

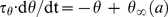
(3)where *a_∞_* is an increasing sigmoidal network input/output function (Table 1). The two parameters *w* and *θ_0_* set the global network excitability [8]. Connectivity (*w*) represents the amount of positive feedback due to excitatory connections, i.e., it determines the fraction of network output (activity) fed back as input to the network. The average cellular threshold (θ_0_) measures the cellular excitability, i.e., it biases the cells' responses to synaptic inputs.

**Table 1:** Parameters of the model.

Parameter	Description	Value
w	Connectivity (synaptic strength)	1 [0.5–3.5]
θ_0_	Input for half maximal activation	0 [−0.2–0.2]
k_a_	Spread of 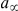	0.05
θ_s_	Activity at half maximal depression	0.3
k_s_	Spread of 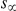	0.05
τ_s_	Time constant for *s*	250 [25–2500]
θ_θ_	Activity at half maximal adaptation	0.3
k_θ_	Spread of 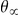	0.05
τ_θ_	Time constant for *θ*	250 [25–2500]
g	Strength of cellular adaptation	1 [0–1.5]

Typical parameter values used in the simulations are shown. For parameters that were varied, the range of values used is also indicated in brackets. The steady state network output function is 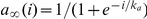, the steady state synaptic availability is 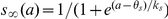 and the steady state activation of cellular adaptation is 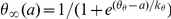.

In Eq (1) we see that synaptic depression, which decreases *s*, acts as a divisive factor, decreasing the amount of positive feedback, while cellular adaptation, which increases *θ*, is a subtractive factor. An additional parameter, *g*, can be adjusted to scale the strength of the adaptation process. Unless mentioned otherwise, *g* is set to 1. The steady state functions *s_∞_* and *θ_∞_* are decreasing and increasing sigmoidal functions of activity, respectively. Thus, when activity is high, *s* decreases and *θ* increases, both of which contribute to active phase termination. During the silent phase, *s* increases and *θ* decreases, eventually initiating a new active phase. The active phase is defined as the period of activity for which *a* is above an arbitrarily determined threshold (0.35). Below this threshold the system is in the silent phase.

The network recruitment time constant, τ*_a_*, is arbitrarily set to 1 and the time constant for the variations of *s* and *θ* are assumed much larger than τ*_a_*. That is, *s* and *θ* are slow processes. All parameter values are given in Table 1. Equations were solved numerically using the 4^th^ order Runge Kutta method (dt = 0.05) in XPPAUT [10]. The simulation code is freely available on RB's website http://www.math.fsu.edu/~bertram/software/neuron.

## Results

**Figure 1:**
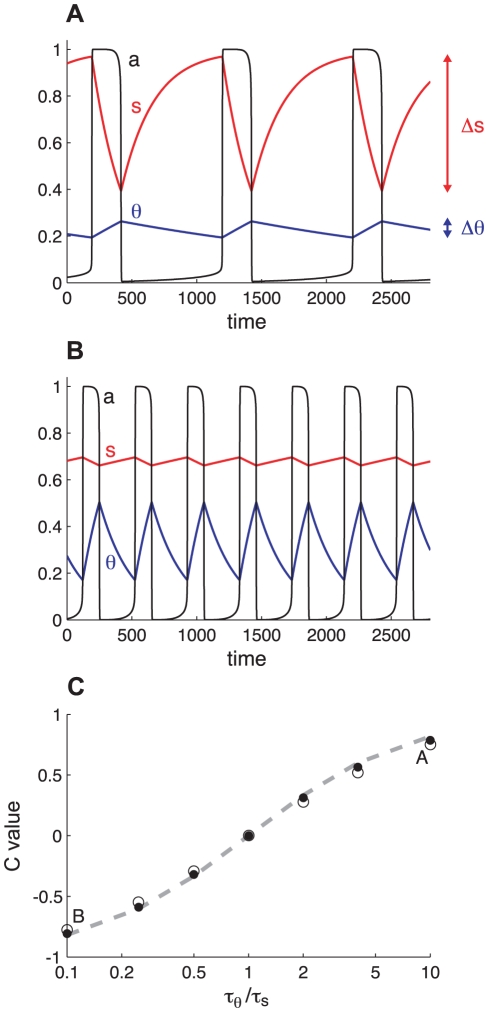
Illustration of the concept that the faster process contributes more to rhythm generation. A. Time courses of activity (*a*, black), the synaptic recovery variable (*s*, red), and the adaptation variable (*θ*, blue) for τ*_θ_*/τ*_s_* = 10. The range of variation of *s* (Δ*s*) is about 10 times larger than the range of variation of *θ* (Δθ). Thus, according to the correlative measure *s* contributes more to the rhythmicity. B. Similar time courses for τ*_θ_*/τ*_s_* = 0.1. The cellular adaptation now appears to contribute more than the synaptic recovery variable. C. Plot of the variations of C =  (R−1)/(R+1) with the τ*_θ_*/τ*_s_* ratio. Closed circles obtained from simulations with θ_0_ = 0; open circles for θ_0_ = 0.18. When *θ* is much faster than *s* (τ*_θ_*/τ*_s_* is small), C is close to -1 indicating that *θ* is the dominant process. When τ*_θ_* ≈ τ*_s_*, C≈0 indicating that both processes have equal contribution to the rhythm. At large τ*_θ_*/τ*_s_*, C approaches 1 and *s* is the dominant process. Points labeled A and B refer to the cases illustrated in panels A and B. Dashed curve, variations of c = (r−1)/(r+1) with τ*_θ_*/τ*_s_*; r = (w/g)(τ*_θ_*/τ*_s_*) and w = g = 1. Results are similar if we keep τ*_θ_*/τ*_s_* = 1 and vary w/g instead.

To assess the contributions of slow divisive and subtractive feedback to episode onset and termination we first test two methods based on measurements and manipulations that can be performed experimentally. We use a mean field model of rhythmic activity in an excitatory neural network regulated by both synaptic depression and cellular adaptation, defined by Eqs. 1–3, to generate synthetic data. These data show the time courses of the network activity, *a*, and the two negative feedback processes, *s* and *θ* (Figure 1AB). When we ask what is the contribution of a process to the episodic activity, we ask two questions: what is its contribution to episode initiation, and what is its contribution to episode termination. To clarify the meaning of “contribution”, we see in Figure 1A or 1B that during an episode *s* decreases and *θ* increases. These effects decrease network excitability and eventually the activity cannot be sustained, so the high-activity episode stops. But which effect is more important in terminating an episode? Was it the decrease in *s* or the increase in *θ*? Can we quantify this notion? Similarly, during the silent phase both processes recover (i.e., *s* increases and *θ* decreases), until a new episode is initiated. Again, can we quantify the effects on episode initiation of the increase in *s* vs. the decrease in *θ*?

### Correlative approach: comparing the variations of the feedback processes

The rationale for this first approach is that if a process varies greatly during the high-activity episodes (active phases) and the inter-episode intervals (silent phases), then it is likely that it contributes significantly to episode termination and onset. On the other hand, if the variations are small, it is likely that the contribution of the process is small. This approach thus relies on observing a relationship between the time course of a process and the system's behavior. Its pitfall, that correlation does not imply causation, is well known.

Experimentally, one can record spontaneous or evoked postsynaptic potentials or currents in target neurons [11], [12], [13], [14]. The variations of this postsynaptic response during the interval of time between two episodes of activity would represent the variations of the effective connectivity, or available synaptic strength, w^.^ *s*. Similarly, one may record the degree of adaptation or the current responsible for this adaptation at various times during the silent phase [11], [14]. The variations of the current with time would be equivalent to the variations of g^.^ *θ*. Here we assume that there are only two slow feedback processes, represented by *s* and *θ*, which can be measured unequivocally and with sufficient precision. This is an ideal situation that will not often be encountered experimentally; we show that even with such ideal conditions we may not be able to determine the contributions of the two slow processes using the correlative approach.

If *s* varies by Δ*s* and *θ* by Δ*θ* over one phase of the oscillation, then according to the correlative approach the ratio 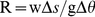
(4)measures the contribution of *s* relative to that of *θ*. We have shown previously [8] that if s and *θ* vary exponentially with time constants τ*_s_* and τ*_θ_*, then Δ*s*/Δ*θ* ≈ τ*_θ_*/τ*_s_* . Thus, 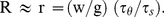
(5)


Assuming that w and g are similar – we set w and g to 1 unless noted otherwise – the ratio of the contributions of the two processes to the rhythmic activity is inversely proportional to the ratio of their time constants, so the faster process contributes more than the slower process. This is illustrated in Figure 1AB where we plot the variations of *a* (network activity), *s* and *θ* for the cases r = τ*_θ_*/τ*_s_* = 0.1 (A) and r = τ*_θ_*/τ*_s_* = 10 (B). In the case shown in Figure 1A, we expect *s* (red curve) to contribute more to episode onset/termination because it is the faster process, while in the case shown in Figure 1B *θ* (blue curve) is faster and thus expected to have the major contribution.

We define a quantitative measure of the contribution of the two processes by 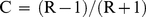
(6)(or, using the approximation given by Eq. 5, c = (r−1)/(r+1)). C varies between −1 and 1. If C is near 1 then *s* determines the episode onset and termination (i.e., *θ* has no role). If C≈−1 then *θ* controls episode onset and termination. Intermediate values of C indicate that both processes contribute. This measure is plotted as a function of r in Figure 1C, and clearly demonstrates the shift of control (according to the correlative definition) from *θ* to *s* as the *s* dynamics are made progressively faster relative to *θ*. The filled circles result from simulations with the cell excitability parameter θ_0_ set to 0 (relatively high cell excitability). The open circles were obtained using θ_0_ = 0.18 (low cell excitability). The differences are very small, showing that, according to this measure, the respective contributions of the two processes depend very weakly on θ_0_. The dashed curve in Figure 1C is obtained by plotting c  =  (r−1)/(r+1). Since the points obtained from plotting C lie almost on this curve, one concludes that, according to the correlative approach, the contributions of the two slow processes depend only on the ratio r  =  (w/g) (τ*_θ_*/τ*_s_*). Thus, the faster that one process is relative to the other the greater its contribution will be to rhythm generation. Similarly, the greater the relative weight of a process, the greater its contribution. Finally, since each process covers the same range during the active and silent phase, these results do not distinguish between episode initiation and termination. That is, the correlative approach predicts that the contribution of each process is the same for episode initiation and termination.

### Blocking approach: deleting one feedback process

The rationale for this second approach is that if a process is important to a system's behavior, then removing it will have a large effect. This type of experiment is widely used in biology and includes pharmacological block, surgical ablation, and gene knockout. If, for example, *θ* represents the activation of a potassium current responsible for cellular adaptation, then one could block this current pharmacologically or genetically and measure the effect on network activity. We block the *θ* process by setting g = 0 and observe the effect on the length of both the active and silent phases after transient effects have died down. If we see a large increase in the active phase duration, then we conclude that this process is important in terminating the active phase. Similarly, if after the block we see a decrease in silent phase duration then we conclude that recovery of this process is important for episode initiation. The pitfall of this approach is that after blocking a process we obtain a different system.

**Figure 2:**
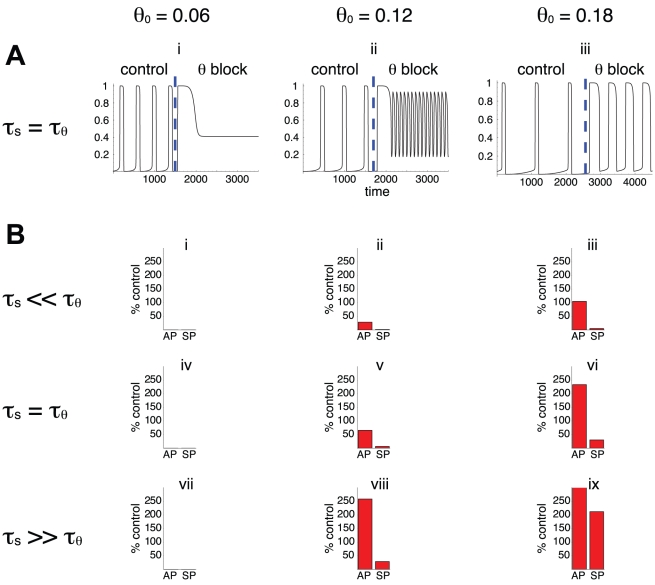
Illustration of the blockade approach. A. time course of network activity before (“control,” g = 1) and after (“θ block,” g = 0) blocking the adaptation process *θ*. These simulations were obtained for τ*_θ_*/τ*_s_* = 1 and for the three values of θ_0_ indicated. Vertical dashed line indicates the time when the process was blocked. B. Effects of blocking *θ* on the lengths of the active and silent phases (AP, SP), represented as percentage of “control”. No bars are shown when rhythmic activity was abolished. There are more cases where *θ* block results in decrease of SP duration (ii, iii, v, vi, viii) than increase in AP duration (vi, viii, ix). The interpretation is that *θ* contributes more to delay episode onset than to provoke episode termination. The results of the blockade experiment depend on the value of θ_0_, the activity threshold in the absence of adaptation, unlike the predictions from the correlative approach (Figure 1C). The blockade experiments also produces similar results in pairs of cases (iii, v) and (vi, viii) that have different τ*_θ_*/τ*_s_* ratio; this is also in opposition to the correlative approach.

Figure 2 illustrates the results obtained with this approach, for different values of the parameter θ_0_. Figure 2A shows the time course of network activity before and after blocking *θ* in the case τ*_θ_* = τ*_s_*. When cell excitability is too high (e.g., θ_0_ = 0.06), synaptic depression alone cannot bring the network to a low activity state and rhythmicity is lost after the block. For lower cell excitability (higher θ_0_, middle and right columns), blocking *θ* leads to changes in the lengths of both the active and silent phases, to various degrees. These changes in active and silent phase durations (AP and SP), after transient effects have died out, are represented on Figure 2B for different values of the ratio τ*_θ_*/τ*_s_*. Can we infer the importance of *θ* variations on rhythm generation from these changes?

We first note that for low θ_0_ rhythmic activity is lost after blocking *θ*, for all values of the ratio τ*_θ_*/τ*_s_*. Thus, variations in *θ* are required for rhythm generation in these cases. In the other cases shown, blocking *θ* has large effects on the active and silent phase durations, but these effects are difficult to interpret. For instance, we expect the block to increase the active phase in proportion to *θ*'s contribution to episode termination. Thus, it seems that *θ* contributes significantly to episode termination in cases vi, viii and ix (where there is a large increase in AP after the block), but does not contribute much to episode termination in case iii (where there is no change in AP after the block). In cases ii and v the active phase duration actually *decreases* after the block, which is hard to interpret. Similarly, we expect the decrease in silent phase duration following *θ* block to be in accordance with *θ*'s contribution to episode initiation, since residual adaptation delays episode onset. Thus, we would say that *θ* contributes significantly to episode onset in cases ii, iii, v, vi and viii. But again, we have an unexpected case (ix) where SP *increases* after the block.

The blockade experiment illustrated in Figure 2 suggests that there are more cases where *θ* has a significant contribution on episode initiation (ii, iii, v, vi, viii) than on episode termination (vi, viii, ix). This is in contradiction with the correlative approach, which suggested that *θ* had a similar contribution to both episode termination and initiation. There are also cases, such as vi and viii, where the effects of the block are similar, suggesting that *θ*'s contribution to episodic behavior is similar in those cases. But cases vi and viii correspond to different values of the ratio τ*_θ_*/τ*_s_*. According to the correlative approach, the contribution of each process should vary with τ*_θ_*/τ*_s_* (Figure 1C), so again the blockage approach and correlative approach disagree. Finally, on each row of Figure 2B the effect of the blockage varies with the value of the parameter θ_0_. This again contradicts the correlative analysis, which showed little dependence on θ_0_.

The strong perturbation to the system effected by the block is responsible for the counterintuitive decrease in AP observed in cases ii, v and increase in SP observed for case ix. These changes reflect system compensation; after the block and after transients have died out, the unblocked process, *s*, covers a different range of values, so AP and SP are modified. This compensation could be avoided by measuring AP and SP just after the block instead of letting it equilibrate. This is illustrated in Figure 2Aii, where the block initially increases AP, then decreases it as SP is decreased by the absence of *θ*. Interpretation of the block experiment would therefore be facilitated by considering only transient behavior, but this would be difficult to do experimentally in most cases. For instance if we block a K^+^ channel pharmacologically then the kinetics of drug application and binding to the channels will interfere with the transient effects.

In summary, we find that the correlative and blockage approaches suggest different interpretations about the contributions of the negative feedback processes to rhythm generation. In the following, we show that neither approach gives a satisfactory description of the contributions of the slow processes. This is because each approach suffers its own pitfall. The first approach is purely correlative, i.e., it links variations in one process to the behavior of the system, but cannot establish causation. To obtain causation it is necessary to determine how the system responds to a perturbation to one of these processes, as in the blocking approach. Unfortunately, by perturbing the system, we change it. The loss of periodic activity after blocking *θ* (as in cases i, iv, vii in Figure 2) shows that this process may be necessary for maintaining rhythmic activity, but it does not indicate what was the contribution of *θ before* the block.

### A new measure of the relative contributions of the negative feedback processes

**Figure 3:**
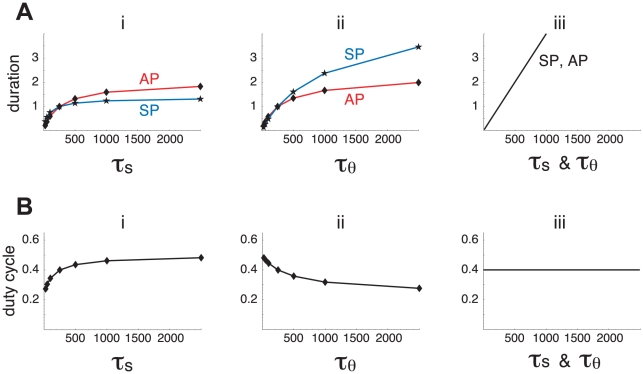
Variations of the time constants τ*_s_* and τ*_θ_* have different effects on the activity pattern. A. Relative change in AP (red, diamonds) and SP (blue, stars) as τ*_s_* (i) or τ*_θ_* (ii) is varied. For comparison, the linear change in both AP and SP when τ*_θ_* and τ*_s_* are varied together by the same factor is shown (iii, τ*_s_* & τ*_θ_*). B. Variations of the duty cycle with τ*_s_* (i), τ*_θ_* (ii) and τ*_s_* & τ*_θ_* (iii).

The goal here is to derive a measure that allows one to draw a causal link between each slow process and the activity pattern that does not involve a strong perturbation to the system. Suppose that *s* is the only negative feedback process regulating episodic activity, so it contributes 100% to both episode termination and initiation. Then doubling τ*_s_* will (approximately) double both AP and SP. If *s* is not the only negative feedback process and therefore has only a partial contribution to episode termination and initiation, then doubling τ*_s_* will still increase AP and SP but by a smaller factor. Thus, the contribution of *s* to the episodic activity can be determined by the fractional change in AP and SP durations following a change in τ*_s_.* To illustrate this idea, we plot both AP and SP durations as either τ*_s_* or τ*_θ_* is varied in Figure 3A.

Figure 3Ai shows that AP varies more with τ*_s_* than does SP. This suggests that *s* has more influence on episode termination than on episode initiation. The variations of AP and SP with τ*_θ_* (Figure 3Aii) show the opposite trend, suggesting that *θ* has more influence on episode initiation than on episode termination. These trends are also illustrated by the variations of the duty cycle ( =  AP/(AP+SP)) with τ*_s_* and τ*_θ_* (Figure 3B). The duty cycle increases with τ*_s_*, but decreases with τ*_θ_*. Finally, comparing Figures 3A i and ii, we observe that the variations of AP with τ*_s_* and τ*_θ_* are similar, suggesting that *s* and *θ* have comparable contributions on episode termination. On the other hand, SP varies more with τ*_θ_* than with τ*_s_*, suggesting that *θ* has a stronger influence on episode initiation than does *s*. This example suggests that the contributions made by the slow processes to the episodic activity can be determined by varying the time constants of the processes and observing the effects on AP and SP durations. We now use this idea to construct a quantitative measure of these contributions.

**Figure 4:**
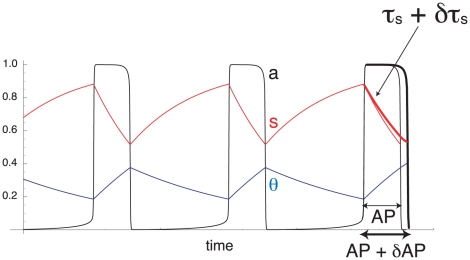
Construction of a measure of the contribution of *s* to episode termination. Increasing τ*_s_* by δτ*_s_* at the beginning of an episode slows down *s* slightly (thick red curve), so the active phase is lengthened by δAP (thick black curve).

We first construct a measure of the contribution of *s* to episode termination, as illustrated in Figure 4. At the beginning of an episode, τ*_s_* is increased by δτ*_s_*. If *s* contributes to episode termination, slowing down *s* increases AP by δAP. We can quantify the contribution of *s* to episode termination by evaluating the ratio of the relative change in AP, δAP/AP, divided by the relative change in τ*_s_*, δτ*_s_*/τ*_s_*. We thus define the normalized contribution of *s* to episode termination as 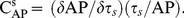
(7)


If *s* has no influence on episode termination, slowing it down has no effect and δAP  =  0. If *s* is the only process contributing to episode termination, then the active phase duration is the time it takes for *s* to decrease from its value at the beginning of an episode to its value at the transition between AP and SP. Since we consider relaxation oscillations, the transition time between active and silent states is negligible. Thus, a fractional change in τ*_s_* leads to the same fractional change in AP (δAP/AP  =  δτ*_s_*/τ*_s_*) so that C^s^_AP_ = 1. Therefore, C^s^_AP_ has a value between 0 (*s* does not contribute to episode termination) and 1 (*s* is the only process contributing to episode termination). We quantify the contribution of *s* to episode initiation similarly using 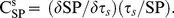
(8)


We define the contributions of *θ* to episode termination and initiation in a similar way:
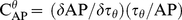
(9)

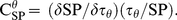
(10)


These measures have the same motivation as the blockage experiment, but can be computed with small perturbations to the system. We use δτ/τ = 4% so the perturbation is small but nevertheless has a measurable effect. In addition, we look at the acute effect of the perturbation, i.e., we do not wait until the system equilibrates.

**Figure 5:**
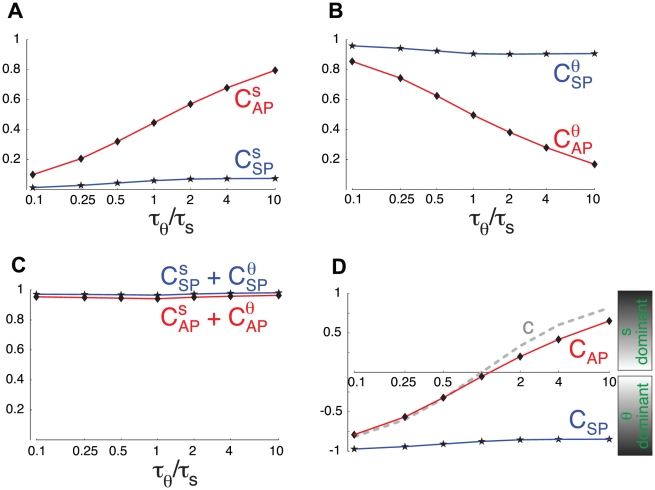
Variations of the contributions of *s* and θ with τ_θ_/τ*_s_*. A. Contributions of *s* to episode termination (C^s^_AP_, red, diamonds) and initiation (C^s^_SP_, blue, stars). B. Contributions of *θ* to episode termination (C^θ^_AP_, red, diamonds) and initiation (C^θ^_SP_, blue, stars). C. Both sums C^s^_AP_ + C^θ^_AP_ (red, diamonds) and C^s^_SP_ + C^θ^_SP_ (blue, stars) are close to 1, demonstrating the consistency of the measures. D. Combined measures C_AP_ (red, diamonds) and C_SP_ (blue, stars), as defined in Eq 11–12, superimposed with the prediction from the correlative measure c (dashed curve, as in Figure 1C). C_AP_≈−1: *θ* controls the active phase; C_AP_≈0: both *θ* and *s* have equal contributions to setting the duration of the active phase; C_AP_≈1: *s* controls the active phase (and similarly for C_SP_ and the silent phase). Variations of C_AP_ show that the relative contribution of *s* to the termination of the active phase increases with τ*_θ_*/τ*_s_*, in agreement with the correlative approach. On the other hand, C_SP_ remains close to -1, showing that the subtractive process (*θ*) controls episode onset over the whole range.

Figure 5A shows the contributions of *s* to episode termination (C^s^_AP_) and initiation (C^s^_SP_) as the ratio τ*_θ_*/τ*_s_* is varied, determined through numerical simulations as shown in Figure 4. C^s^_AP_ increases as this ratio is increased, that is, *s* contributes more to episode termination as it becomes faster relative to *θ*. When *s* is much slower than *θ*, C^s^_AP_ is close to 0. For *s* much faster than *θ*, C^s^_AP_ is close to 1. When *s* and *θ* have similar speed C^s^_AP_ is close to 0.5, suggesting that the divisive and subtractive feedback processes contribute equally to episode termination when their time constants are similar. This relationship between the contribution of feedback processes to episode termination and the ratio of their time constants is in agreement with the prediction from the correlative approach (Figure 1C). However, the contribution of *s* to the silent phase, C^s^_SP_, varies differently with τ*_θ_*/τ*_s_*. Although it increases with τ*_θ_*/τ*_s_*, this increase is so weak that C^s^_SP_ is below 0.1 even if *s* is 10 times faster than *θ*. This consistently low C^s^_SP_ suggests that regardless of the relative time constants of the two negative feedback processes, *s* never contributes significantly to episode onset, in sharp contrast with the prediction from the correlative approach.

Figure 5B shows that the contributions of *θ* to episode termination (C^θ^_AP_) and initiation (C^θ^_SP_) vary in the opposite way to C^s^_AP_ and C^s^_SP_. If τ*_s_* is much larger than τ*_θ_* then *s* does not affect AP while *θ* strongly affects AP. As the ratio τ*_θ_*/τ*_s_* increases, the contribution of *s* to episode termination increases while the contribution of *θ* decreases, in such a way that the sum of the contributions of *s* and *θ* stays around 1 (C^s^_AP_ + C^θ^_AP_ ≈ 1) as shown in Figure 5C. The effect of *θ* on SP is always strong, while the effect of *s* is weak, regardless of τ*_θ_*/τ*_s_*. The sum of the contributions of *s* and *θ* to episode initiation also stays around 1 (C^s^_SP_ + C^θ^_SP_≈1). Thus *s* and *θ* have complementary contributions to the episodic activity and our measure is self-consistent. The relationship C^s^_xP_ + C^θ^_xP_≈1 is a consequence of the fact that *s* and *θ* are the only processes controlling AP and SP. That is, if we increase both of their time constants by a factor k, then AP and SP both increase by the same factor k (Figure 3Aiii). This can be written, in the case of the active phase, as: AP(k τ*_s_*, k τ*_θ_*)  =  k AP(τ*_s_*, τ*_θ_*). Application of Euler's theorem for homogeneous functions yields: 
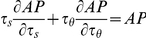
 and, after dividing each side by AP, results in C^s^_AP_ + C^θ^_AP_ = 1.

Since we are dealing with only two slow processes, we can combine the measures defined for *s* and *θ* (Figures 5A and 5B) into single measures by defining
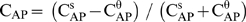
(11)

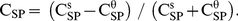
(12)


With this definition, C_AP_ and C_SP_ vary between −1 to 1. A value close to −1 signifies that *θ* is the dominant process; a value close to 1 signifies that *s* is the dominant process; a value near 0 means that *s* and *θ* have similar contributions. These are plotted in Figure 5D as a function of τ*_θ_*/τ*_s_.* We see that C_AP_ rises from −1 to near 1 as τ*_θ_*/τ*_s_* increases, indicating that *θ* dominates the AP when it varies more rapidly than *s*, and *s* dominates when it varies more rapidly than *θ*. This agrees with the result obtained with the correlative approach (dashed curve, c =  (r−1)/(r+1)). In contrast, the SP is controlled by *θ* for the full range of τ*_θ_*/τ*_s_*; this was not predicted by the correlative approach.

### Conditions of applicability of the measure

The contribution measures defined above are meaningful only if specific conditions are satisfied. The most important condition is that each variable or process contributes to the same aspect of system behavior. For instance we cannot compare the contribution of a slow negative feedback process, such as our *s* or *θ*, which terminates an episode of activity, to the contribution of a fast negative feedback variable that could be responsible for fast cycling during the high activity phase. Second, the variables must vary monotonically during each phase of the activity. If not, then increasing their time constant may not increase the duration of a phase in a predictable way and the sum of the contributions of the variables to that phase may not equal 1.

We use a relaxation oscillator with a clear distinction between active and silent phases. The measure can be applied to other types of oscillations, as long as active and silent phases can be clearly distinguished. In more complex cases, it may be necessary to divide a period of activity into more than two phases. More generally, the method could be applied to non-oscillatory systems, for example to determine the contribution that different variables make to return the system to an equilibrium following a perturbation. Also, the measure is not limited to two negative feedback processes. We have chosen feedback processes of different types, subtractive and divisive, because we find the problem of disentangling their relative contributions to be quite challenging. This measure can be applied with feedback processes of the same type, as long as they contribute to the same behavior. We have used the method to compute the respective contributions of two subtractive feedback processes to burst generation and shown that the results can be used to predict the occurrence of phase-independent resetting [15]. Finally, we use a deterministic model. Noise would not qualitatively affect our measure, as long as it does not affect the mechanisms for the transitions between phases. If noise is part of the transition mechanism [16] our method cannot be applied as it is, since noise would also contribute to the transitions.

Since the measure requires a model of the system, the validity of its results depends on the validity of the model. Models may incorporate various degrees of realism, so it is important that the measure be robust to model details. For instance, if we add a fast variable to the relaxation oscillator model, so that fast oscillations (spikes) are produced during each active phase, the two slow negative feedback processes may still terminate episodes (bursts) like in the relaxation case. Thus, the relative contributions of each slow variable to burst onset and termination should not change qualitatively. We have demonstrated such robustness with a model of bursting in pancreatic islets [15].

### The effect of network excitability on the contributions of s and θ

**Figure 6:**
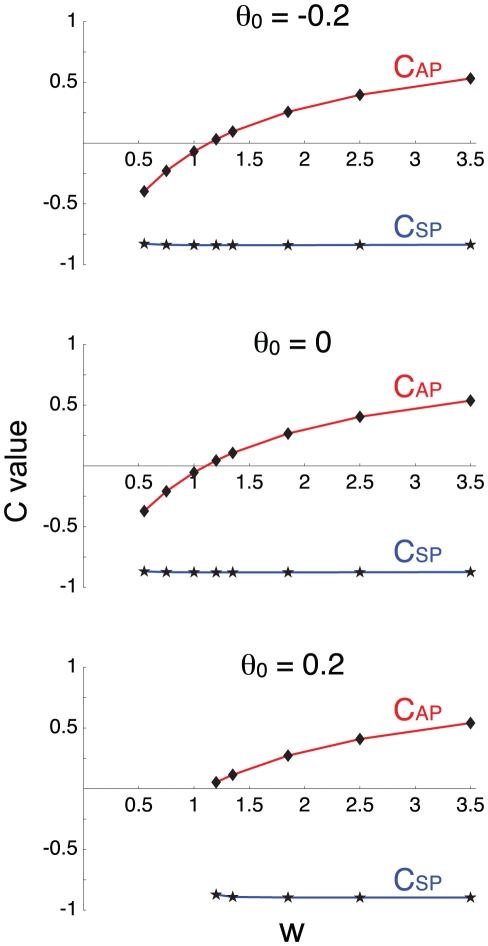
Variations of C_AP_ and C_SP_ with network connectivity and cell excitability (for τ_θ_/τ*_s_* = 1, g = 1). C_AP_ increases with increased synaptic connectivity, as would be expected from the correlative measure (Eq 4), with equal contributions from both processes (C_AP_ = 0) when (w/g) (τ*_θ_*/τ*_s_*)  = 1. In contrast, C_SP_ is always close to -1, the subtractive feedback process sets the length of the silent phase regardless of the value of w. Finally, both C_AP_ and C_SP_ are unaffected by changes in θ_0_, showing that cell excitability does not influence which process controls episodic activity.

We now evaluate how the parameters that control network excitability, *w* (network connectivity) and *θ_0_* (average cellular threshold), affect the contributions of *s* and *θ* to rhythm generation. Variations of C_AP_ and C_SP_ with *w* are represented in Figure 6, for three different values of *θ_0_* (and for τ*_θ_*/τ*_s_* = 1). Clearly, C_AP_ increases with *w*, i.e., synaptic depression contributes more to episode termination when network connectivity is high. However, this is not true for episode initiation, as C_SP_ is almost unaffected by *w*. There is in fact a slight tendency for C_SP_ to increase at the lowest values of *w*, which is more visible if *s* is faster than *θ* (not shown). Changes in *θ_0_* do not affect either C_AP_ or C_SP_ significantly. This is in agreement with the correlative approach, but in contrast to the results of the blockade experiment (Figure 2).

In summary, the ratio τ*_θ_*/τ*_s_* and connectivity *w* – but not *θ_0_* – strongly affect C_AP_, while none of these have a significant effect on C_SP_. In general, both feedback processes *s* and *θ* play roles in the episode termination, but only *θ* controls episode initiation. The relative influence of *s* and *θ* to episode termination varies with parameter values. The correlative approach is roughly correct for predicting the contributions of the two processes to episode termination, but not to episode initiation. This approach makes a direct comparison between the time scales of the two processes, scaled by their relative strength (w and g), evaluating r = (w/g) (τ*_θ_*/τ*_s_*). But this ratio is not the ratio of the contributions of the two processes to episode initiation. In fact, we show below that the weighted time scales cannot be compared directly but must be rescaled, the correct ratio being
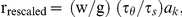
where the scaling factor *a_k_* is the activity level at the transitions between active and silent phases. At episode termination, *a_k_*≈1 so the correlative approach is approximately right. However, at episode onset *a_k_*≈0, so r_rescaled_ ≈ 0, meaning that *s* does not contribute significantly unless r >> 1. Looking back at Eq. 1, it is evident that *s* generally has little effect when activity is low. Such a simple fact was not revealed using the correlative and blockade approaches, stressing again that these standard experimental approaches are not always useful for determining the contributions of different variables to rhythmic activity.

### Effects of g and problems of the blockade experiment

**Figure 7:**
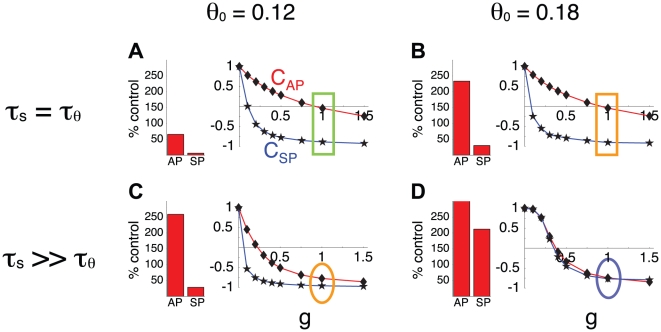
The blockade experiment does not inform on the relative contributions of each process to rhythmic activity. Panels A, B, C, D correspond to cases shown in panels v, vi, viii, ix in Figure 2. For each case, the change in AP and SP durations following *θ* block is shown next to the variations of C_AP_ (red, diamonds) and C_SP_ (blue, stars) with g (the maximum amplitude of cellular adaptation). Blocking *θ* means changing g from 1 to 0. As g reaches 0, both C_AP_ and C_SP_ reach 1 since *s* becomes the only variable controlling episodic activity. In A and B, the contributions measures are also the same before the block (g = 1, rectangle highlights), nevertheless the blockade leads to different changes in AP and SP durations. Thus, these changes in durations after the block cannot be used to predict the respective contributions of each process before the block. Panels C and D illustrate the same points, with similar contributions measures before the block (oval highlights) but different effects of the block on AP and SP durations. Finally, panels B and C show that despite different contributions measures (oval vs. rectangle highlight) before the block, the resulting effect of the block on AP and SP durations are the same. Again, results from the block do not provide much information about the respective contributions of each process before the block.

The analysis above suggests that the correlative approach can reasonably estimate the contribution of each process to episode termination, but misses the fact that *s* contributes little to episode onset (Figure 5D). Results from both the blockade simulations and the analysis above suggest that *θ* is more important for episode initiation than episode termination. However, we have seen that the blockade approach does not typically provide a good indication of the contribution of *θ* to the AP and SP durations (Figure 2B). To further demonstrate this, we plot in Figure 7 the variations of C_AP_ and C_SP_ with g (curves), the maximal “conductance” of the adaptation process *θ*, in four of the cases illustrated in Figure 2B (v, vi, viii, ix). The values of both C_AP_ and C_SP_ decrease as g is increased, indicating that the influence of *θ* in the control of the rhythm increases with g. As g decreases towards 0, both C_AP_ and C_SP_ increase toward 1 since *s* is the only slow process when g = 0. This is true for all four cases. However, C_SP_ only increases noticeably when g approaches 0, illustrating again that the subtractive feedback process controls the silent phase in most cases.

Comparing Figure 7A–B, we see that the C_AP_ curve is similar in both panels, as is the C_SP_ curve. The bar plots show the effects of a blockade simulation, where g = 1 before the blockade and g = 0 afterwards. In Figure 7A the blockade results in a 50% reduction in the AP duration, while in Figure 7B there is a very large increase in the AP duration following blockade. Yet, according to the C_AP_ curves the contribution of *θ* to the AP duration is nearly the same in both cases when g = 1 (green and yellow boxes). Similarly, C_SP_ is similar in panels C and D for g = 1, yet the blockade results in decreased SP duration in C, but increased SP duration in D. Thus, the effects of the blockade on AP and SD durations do not provide much information on the respective contributions of the two processes before the blockade.

Next, we compare cases shown in Figure 7B and 7C. We notice that C_AP_ differs between the two cases, showing that when g = 1 the *s* variable contributes significantly to episode termination in one case (Figure 7B) but not the other (Figure 7C). Yet, after blockade the changes in AP/SP (bar plots) are similar in both cases. Again, results from the blockade approach do not indicate what was the contribution of each process before the blockade.

### A geometric measure of the relative contributions of the negative feedback processes

**Figure 8:**
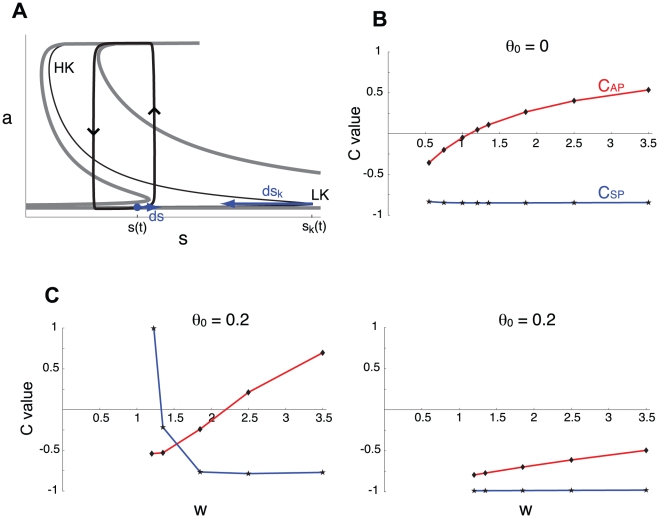
Alternate estimation of the relative contributions of each process using phase plane analysis. A. Representation of the system in the *a*,*s*-plane. The system trajectory is shown as a thick black curve with arrows at the transitions between activity phases. The trajectory follows the dynamic nullcline (thin black S-shaped curve) which moves left during the silent phase and reaches the thick gray nullcline on the left at episode onset. At onset, the trajectory reaches the low knee (*s*(t) = *s*_k_(t)). During the active phase, the dynamic nullcline moves to the right toward the thick gray nullcline on the right. It reaches it at episode termination, as the trajectory reaches the high knee. Note that the lower portion of the nullcline is much more sensitive to *θ* than the higher portion. LK, low knee; HK, high knee of the a-nullcline. B. Variations of C_AP_ and C_SP_ (calculated using the phase plane approximation illustrated in A) with *w* (for τ*_θ_*/τ*_s_* = 1 and θ_0_ = 0). There is good agreement with the computational method based on small perturbations in the time constants (compare with Figure 6B). C. For large values of θ_0_, such that high activity episodes require that *θ* be close to 0, the computational calculation of the relative contributions (left panel) and the phase plane estimation (right panel) can disagree. In the case shown, τ*_θ_*/τ*_s_* = 0.1 (w = g = 1), so the phase plane method estimates that *θ* should control both active and silent phase (right panel). The disagreement with the computed C_AP_ and C_SP_ (left panel) is a result of the geometric argument used to estimate the contributions neglecting the fact that the speed of variation of *s* and *θ* can slow down dramatically when approaching their asymptotic values (see text).

For the mathematically simple system used in this work, we can use a geometrical argument to derive approximate formulas for C_SP_ and C_AP_. If the system is two-dimensional with one slow process, *s*, the trajectory could be drawn in the *a*,*s*-phase plane and would follow the *a*-nullcline (except for fast jumps at the transitions between active and silent phase). For the three-dimensional system presented here, the trajectory in the three-dimensional *a*,*s*,*θ*-phase space follows the surface defined by d*a*/d*t* = 0 [8]. We can project the three-dimensional trajectory and surface into the *a*,*s*-plane. This results in a two-dimensional trajectory that follows a *dynamic a*-nullcline (Figure 8A). The effect of the third variable (*θ*) in this two-dimensional representation is to move and deform the dynamic *a*-nullcline (the thin, black S-shaped curve in Figure 8A). Increasing *θ* moves the nullcline rightward.

At the end of the active phase, the trajectory falls from the high- to the low-activity state and the dynamic nullcline is at its rightmost position (thick, discontinuous, grey S-shaped curve on the right of the diagram). During the silent phase, *s* increases so the system's trajectory moves to the right while *θ* decreases so the a-nullcline is transformed leftward. When the trajectory passes the low knee (LK) of the nullcline, the trajectory jumps to the upper branch. At this point the nullcline has reached its leftmost position (the thick grey S-shaped curve on the left), since *θ* will now again begin to increase and the *a*-nullcline will be transformed rightward.

To compare the contributions of *s* and *θ* to the termination of the silent phase, we can therefore compare the length traveled by the trajectory (controlled by *s*) with the length traveled by the low knee (controlled by *θ*). Assuming that their speeds are nearly uniform, we can compare the instantaneous variation of the trajectory's position d*s* to the instantaneous variation of the knee d*s_k_* due to the variation of *θ*, d*θ*. We can show [8] that d*s* ≈ d*θ* (τ*_θ_*/τ*_s_*) and that d*s_k_* ≈ (g/w) (d*θ*/*a_k_*) where *a_k_* is the activity level at the knee (its value varies little with *θ*). Thus, the ratio of the contributions of *s* and *θ* is 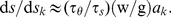
(13)


This formula applies to both active and silent phases, however the activity level at the knee, *a_k_*, differs between the two phases. During the silent phase, *a_k_* is close to 0 so d*s*/d*s_k_* is very small, i.e., *s* generally contributes little to the termination of the silent phase. On the other hand, during the active phase *a_k_* is close to 1, so d*s*/d*s_k_* ≈ (τ*_θ_*/τ*_s_*) (w/g). If (τ*_θ_*/τ*_s_*) (w/g) ≈1 then the two slow processes contribute similarly to active phase termination. This shows that the relative contributions of *s* and *θ* are qualitatively different for the different phases of activity. It also explains why the intuitive approach illustrated in Figure 1 is correct for the active phase (where *a_k_*≈1), since from Eq. 5 and Eq. 13 r ≈ d*s*/d*s_k_*. If (τ*_θ_*/τ*_s_*) (w/g) ≈ 1, then r ≈ 1 and the correlative approach predicts equal contributions of the feedback variables (Figure 1C). On the other hand, during the silent phase *a_k_*≈0 so r is not a good approximation to d*s*/d*s_k_* and the correlative approach is invalid.

To compute d*s*/d*s_k_* for both phases, we must compute *a*_k_ (Eq. 13) for both knees of the dynamic a-nullcline shown Figure 8A. For this we note that the nullcline is defined by d*a*/dt = 0. Solving for *s*, we obtain
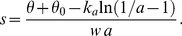
(14)


For each value of *θ*, the knees are defined by 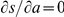 and differentiating Eq. 14 gives:
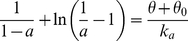
(15)which has two solutions *a*_k_, each corresponding to a knee, provided the right hand side is greater than 2. The values of *θ* at onset and termination of the episodes, to be used in Eq 15, were obtained from the durations of the active and silent phases obtained from simulations [8]. Finally, when *θ*_0_ is changed there is a similar but opposite change in the range of variation of *θ*, so *θ* + *θ*_0_ is not affected much by a change in *θ*_0_. Thus the solutions of Eq. 15 are not very sensitive to *θ*_0_. This explains why the relative contributions of *s* and *θ* are little affected by *θ*_0_, as seen in Figure 6.

Since we identify d*s*/d*s_k_* to the ratio of the contribution of the two slow variables for each phase, C^s^_xP_/C^θ^_xP_, the combined measures C_xP_ defined in Eq 11–12 correspond to the ratios (d*s*/d*s_k_* − 1)/(d*s*/d*s_k_* + 1). These ratios are computed for both active and silent phases as a function of w and shown on Figure 8B. Comparison with Figure 6 (middle panel) shows that this geometric measure of the contributions of the slow processes is in good agreement with the empirical measure constructed above using sensitivities to the slow variables' time constants.

Finally, we point out that there are rare situations when the two measures (Eqs. 7–12 vs. Eq. 13) do not give similar results. Such a case is shown in Figure 8C, for which the parameter *θ*_0_ is large (average cell excitability is low) and τ*_s_* is 10 times greater than τ*_θ_*. Because *θ*_0_ is large, even when *θ* decreases to its minimum during the silent phase, *s* may not be sufficiently large for an episode to start, particularly if the connectivity is low. In that case, an episode is not started until *s* reaches the value corresponding to the low knee. Even if this is a small distance, it can take a long time since *s* is so slow. Thus, changing τ*_s_* can have a strong effect on the silent phase and C_SP_ determined from Eq. 12 becomes positive (Figure 8C, left panel) instead of close to -1 as computed using Eq. 13 (Figure 8C, right panel). In other words, using a measure based on time indicates a strong contribution of *s* in that particular situation, while a measure based on geometry indicates a marginal contribution of *s* to episode initiation. This discrepancy between the two measures appears because *θ* does not vary uniformly. It slows down considerably as it approaches its asymptotic value, “waiting” for *s* to reach the low knee. Thus the dynamics of *s* now play a major role in terminating the silent phase. Note that *θ* still has a strong effect on the *s* dynamics during the silent phase (it determines the location of the low knee of the a-nullcline in Figure 8A), but *θ*'s *dynamics* do not affect the silent phase duration much, so the measure that relies on perturbing the time constants finds it has little contribution.

## Discussion

Biological systems are characterized by the interactions between many components. Often, several processes contribute to regulate the same behavior. The purpose of this work was to develop a method for determining how two different negative feedback processes contribute to the generation of relaxation oscillations in biological systems such as excitatory neuronal networks. We gave a precise meaning to the contribution of a given process to episodic activity in an excitatory network regulated by two activity-dependent negative feedback processes. Namely, a process contributes significantly to the termination of a phase (active our silent) of the activity if an acute change to its time constant at the beginning of the phase significantly lengthens that phase. To illustrate this concept we have used a mean field model of an excitatory neuronal network in a relaxation oscillation regime, regulated by two types of negative feedback, divisive (synaptic depression) and subtractive (cellular adaptation). The measure developed here shows that there is differential control of the two phases by the two feedback processes. Both divisive and subtractive feedback processes contribute similarly to episode termination, as long as their time constants and strengths (i.e., associated conductance) are in the same range. In contrast, only the subtractive feedback process contributes significantly to episode initiation in most cases. This difference in the control of the active and silent phases arises from the very nature of the divisive feedback: acting as a multiplicative factor to the activity level, its influence is much lower during the silent phase when activity is low. Thus during the silent phase the dynamics of the subtractive process play a larger role.

### Experiments alone might not determine the relative contributions of the slow processes

We have first attempted to use approaches inspired from experimental methodology to determine the relative contributions of the two feedback processes to rhythm generation. These included comparison of the time course of each process (the correlative approach) and blocking one of the processes.

The correlative approach simply compares the amount of variation of each process, scaled by each process' strength or conductance. Since the two processes vary by the same amount during the active and silent phase, this approach does not distinguish between active and silent phase. According to this approach, the relative contribution depends only on the ratio of their time constants (τ*_θ_*/τ*_s_*) and on the ratio of their strength (w/g). It predicts that if these two ratios are close to 1 then both feedback processes contribute similarly to the rhythm. In the example shown here this is a good approximation for the active phase. However, for the silent phase, this intuitive rule fails, because an additional scaling factor must be introduced to compare the contributions of the two different negative feedback types. This scaling factor is significantly different from unity for the silent phase; it reflects the fact that the divisive feedback process, being a multiplicative factor to the activity, has very little effect at low activity (i.e., during the silent phase).

The blockade approach suggests that the subtractive process might be more important in setting the silent phase duration, since blocking this process affected the silent phase duration more often than the active phase duration. In this way it provides a piece of information that is missed by the correlative approach. However, similar effects of the blockade on AP and SP durations were found in cases where the ratio of time constants was different (and different effects when that ratio was identical), contradicting the correlative approach and, as shown in Figure 7, contradicting our measure of the relative contributions of each process. Furthermore, unlike the correlative approach, the blockade experiment suggests a strong effect of θ_0_ (which biases the input/output relationship of the system). In general, however, this parameter has little effect on the contribution of each process (cf. Figure 6).

These disappointing results from the two experimental approaches are due to their well known pitfalls: passive observation only establishes an association without proving a causal relationship, while perturbations to the system, such as blockade experiments, can qualitatively change the system being studied. The use of total blockade may be considered extreme. A partial block can potentially be more informative than a complete block because a small enough perturbation may indicate a trend in a component's influence and preclude switching the system to a different mode of operation (see e.g., [17], [18], [19]). In other words, if the perturbation is small enough the effect on the activity may be close to linear so the effect of the partial block can be quantified and provide information on the role of the process that is partially blocked. However, partial blockade cannot provide a quantitative measure with the properties (summation to 1) of the C values developed here.

Our approach, instead, is to use small perturbations to the time constants of the feedback processes and look at the effect immediately following the perturbations. This minimizes the perturbation to the system, while quantifying the relative contribution of the two slow processes to the rhythmic behavior. This method could be applied to many oscillatory systems that rely on the interplay between positive feedback and several negative feedback processes. However, for most known experimental conditions, this method seems impossible to implement. To apply the method requires 1) the ability to change the time constants of the variables of interest one by one, 2) these changes must remain small but have measurable effects and 3) the system's behavior immediately after the changes must be measured, without waiting for transients to die out. For example, in the context of a neural network, there is currently no technique available to change the time constant of synaptic depression by a small amount, quickly and without affecting other network parameters. Thus, in many cases, the question of determining the contributions of different negative feedback processes in rhythm generation (using our approach) may only be addressed with computational models.

One example in which our approach could be used in an experimental setting is the electrical oscillatory activity of single cells. The mathematical formalism used to describe the mean activity of an excitatory network is similar to the Hodgkin-Huxley formalism commonly used to describe the electrical activity of excitable cells [8], [20], [21], [22]. In excitable cells, the sodium or calcium channels generate voltage-dependent inward current, providing fast positive feedback that increases membrane potential, while the delayed activation of outward potassium currents and inactivation of the inward currents provide negative feedback. An outward current has an opposite influence to the excitatory inward current and therefore provides subtractive feedback; on the other hand the inactivation of an inward current is a multiplicative term reducing the amount of positive feedback and therefore is a divisive feedback process. Preliminary results with the Hodgkin-Huxley model of nerve excitability [20] in a repetitive spiking mode suggest that while both sodium current inactivation and potassium (K^+^) current activation contribute to terminating an action potential, it is mostly the de-activation of the K^+^ current that initiates the next spike (J. Tabak, unpublished results). This could be verified experimentally for electrically compact cells using the dynamic clamp technique, which allows one to introduce a model-generated ionic current into a cell [23], [24]. For example, one could pharmacologically block the Na^+^ current, then re-introduce it into the cell using the dynamic clamp. Because the added current is computed from a model, it would be possible to change its inactivation time constant by a desired amount and measure the effect of this perturbation on the duration of the spike or interspike interval. To our knowledge, a similar experiment has been done only once, to show that increasing the inactivation time constant of a low-voltage-activated calcium current would result in longer bursts in invertebrate neurons [25]. While both divisive and subtractive feedback can in principle terminate bursts in neurons [26] it is usually the latter that is considered to regulate bursting, in the form of slow, calcium-activated K^+^ currents. The experiment described in [25] provided strong support for a role of low-voltage-activated calcium current inactivation (divisive feedback) in burst termination.

### Other analysis techniques

Modeling is being established as an essential tool for understanding complex biological systems [27], complementing experimental approaches. But more than mere simulations of systems of differential equations, which are akin to experiments, it is the qualitative analysis of the models that provides new insights into a system's dynamics. Qualitative model analysis techniques include phase plane and bifurcation analysis, but these techniques become more difficult to apply as the number of variables increases. The commonly used fast-slow analysis, which simplifies model analysis by formally separating the equations into fast and slow subsystems, may have limited usefulness when many variables operate on the same time scale.

An extension of fast-slow analysis that can deal with many variables operating on the same time scale is the Dominant Scale Method (DMS) [28]. This method follows one variable of interest along an oscillatory trajectory (for instance, voltage in a cellular oscillator model) and determines the sensitivity of this variable at each point on its trajectory to other variables that are present in its differential equation. During different epochs of time, only a few variables may significantly affect the primary variable, so the model can be reduced to a few variables during each epoch. Thus, a complex model is transformed into a sequence of simpler models using only the dominant variables, and qualitative analysis of the dynamics is possible for each successive epoch [29]. The DMS can evaluate the relative contributions of variables that have different roles, unlike the measure presented here. However, our approach uses the sensitivity of observable features of the system behavior (AP and SP), not the sensitivity of a variable to other variables. For this reason, one may use our approach to identify cases where a variable has very little effect on the primary variable but nevertheless controls the duration of a given phase of the activity (as discussed in last section of Results).

Our approach to measure the contribution of feedback processes to rhythmic behavior is to compute the sensitivity of the AP and SP to the time constants for these processes. Other techniques that use sensitivities of observables of a system to control parameters are Metabolic Control Analysis and Biochemical Systems Theory [30], [31], which have been used to analyze metabolic and gene regulatory networks. Important features of these approaches include summation theorems, for instance the sum of the sensitivities of the level of a metabolite to control coefficients is equal to 1. A similar summation theorem holds in our analysis, where the contributions of the two slow variables to the AP or SP duration sum to 1. These techniques are usually applied to the control of steady states, but they have also been used to describe how observables such as the period and amplitude of an oscillatory system are regulated by control parameters [32], [33]. The control of these observables is usually distributed across control parameters [33]. Here, we found that the control of the active phase is distributed across the divisive and subtractive feedback processes, but control of the silent phase is mostly operated by the subtractive process, *θ*. That is, *θ* is the “rate limiting factor” in the termination of the silent phase.

Finally we mention parameter search techniques, which are usually developed to find parameter sets that lead to a target behavior. These techniques can also be used to determine what parameter changes must be done to qualitatively affect a system's activity and provide information about the robustness of such activity [34]. Furthermore, by finding different parameter sets that produce similar system behavior, it is possible to determine the relationships between parameters that allow a behavior to be maintained [35] or to evaluate how each model parameter influence a given characteristic of the behavior using nonlinear regression [36]. This “database approach” indirectly provides information about the role played by some variables of the system and how a variable can take over when another variable is eliminated. It can be used to explore the behavior of a model in different regions of parameter space [37]. An intriguing observation is that different parameter combinations in a wide area of parameter space may produce similar oscillatory patterns [38]. If two distinct parameter sets produce the same system behavior, does this mean that a variable might have different roles in different networks that produce similar activity? This question could be answered with a combination of the database approach and the analysis technique developed here.

### Conclusion

We have developed a computational method to quantify the relative contributions of feedback processes to active and silent phases of episodic activity. We have considered a case involving both subtractive and divisive processes. If both processes have similar strength and time scales, they contribute equally to terminate the active phase. This is consistent with our intuition and predicted by the correlative approach. Interestingly, it is the recovery from the subtractive process that sets the duration of the silent phase. This is because the divisive feedback is a multiplicative factor to the system's activity and therefore plays little role during the silent phase. Thus, different phases of the activity are controlled differently by the negative feedback processes. Experimental methodologies do not in general provide this type of information, so the determination of the relative contributions of different variables to a biological system's activity will usually require the development of a computational model. The method presented here can be applied to a wide array of oscillatory systems.
